# The Aetiology of Olfactory Dysfunction and Its Relationship to Diet Quality

**DOI:** 10.3390/brainsci10110769

**Published:** 2020-10-23

**Authors:** Richard J. Stevenson, Mehmet K. Mahmut, Annette Horstmann, Thomas Hummel

**Affiliations:** 1 Department of Psychology, Macquarie University, Sydney, NSW 2109, Australia; mem.mahmut@mq.edu.au; 2 Department of Psychology and Logopedics, Faculty of Medicine, University of Helsinki, 00290 Helsinki, Finland; annette.horstmann@helsinki.fi; 3 Interdisciplinary Smell and Taste Center, Department of Otorhinolaryngology, Medical Faculty Carl Gustav Carus, TU Dresden, Fetscherstrasse 74, 01307 Dresden, Germany; thomas.hummel@tu-dresden.de

**Keywords:** olfactory dysfunction, anosmia, aetiology, diet, Western-style diet, diet change

## Abstract

People with olfactory loss may choose foods rich in sugar, salt and fat to compensate their loss—foods that constitute a Western-style diet (WSD). However, olfactory dysfunction has not been consistently linked to any particular type of dietary change. Here we considered whether the aetiology of olfactory dysfunction may affect consumption of a WSD. Two-hundred and twenty-two people with olfactory dysfunction of varying cause, were tested for chemosensory performance and their frequency of consumption of a WSD. There was no evidence of a link between a WSD and olfactory dysfunction at the aggregate level, but an aetiology-based approach revealed various patterns, showing both positive and negative associations between olfactory performance and consumption of a WSD. We suggest a number of reasons why, in certain cases, greater olfactory dysfunction may be linked to lower intakes of a WSD, and the role that different aetiologies may have in affecting choices for foods that may appeal following olfactory impairment.

## 1. Introduction

Olfaction is a core component of flavour perception [1]. It is necessary for evaluating food prior to consumption via the orthonasal route (sniffing), and once in the mouth, retronasal olfaction, along with gustation and somatosensation, provide the sensory input, which gives food its flavour and makes eating pleasurable [2]. Not surprisingly then, olfactory dysfunction can significantly impair flavour perception (e.g., [3,4,5]), reduce the pleasure associated with eating and drinking (e.g., [6,7,8,9]) and negatively impact a person’s quality of life (e.g., [9,10]). While there is universal agreement that loss of olfaction reduces food enjoyment, its specific effects on food choice, and relatedly the nutritional quality of the diet, have been far more varied. In this report we examine if one putative source of this variation could be the aetiology of olfactory impairment, something that has received little attention in previous investigations.

Reports of dietary change consequent of olfactory loss (or impairment) are common. Ferris and Duffy [11] found that two-thirds of their olfactory dysfunction sample reported dietary change, but there was little evidence for what this dietary change entailed. If olfaction is impaired, one might expect some sensory compensation by choosing foods where the predominant sensory impact was gustatory (i.e., sugar, salt) or somatosensory (i.e., fat, irritants). This would favour discretionary processed foods, which are typically rich in added sugar, salt and saturated fat, and comprise a “Western-style” dietary pattern. A shift to a Western-style dietary pattern, has been associated with weight gain in population studies, but with olfactory dysfunction evidence for weight gain is equivocal. Almost as many studies provide evidence of weight loss following olfactory dysfunction (e.g., [12,13]) as they do of weight gain [11,13].

Several studies have examined reports of what people with olfactory dysfunction eat or what has changed in what they eat since their dysfunction arose. Here too, the evidence is mixed. Aschenbrenner et al. [14] reported that some people just ate less overall, while others consumed fewer sweets, and sweet and fatty foods (like cake), but more fruit and vegetables. Zang et al. [5] also reported reduced consumption of sweet and salty food, and Kong et al. [15], reduced fat intake, suggesting a shift to a healthier diet. In contrast, Keller and Malaspina [16] reported highly idiosyncratic changes in diet, while Menesse et al. [6] found higher condiment use and more sugar and cream, with no change in preference for fruit, vegetables and meat. Miwa et al. [9] reported a reduction in fresh fruit and fish consumption in a Japanese olfactory dysfunction sample, and an increased liking for spicy food. While cultural factors may be one source of variation (e.g., prevalence of dietary irritants; [4]), the nature of the olfactory dysfunction may also be important [17].

Olfactory dysfunction has many causes, but the most frequently encountered (e.g., [18,19]) apart from aging [20], arise from sino-nasal disease, post-infection, head trauma, congenital causes, idiopathic loss (i.e., no clear cause) and from a number of miscellaneous causes (e.g., toxins, drugs, iatrogenic). There are several reasons to suspect that different aetiologies may affect diet quality in diverse ways. First, sino-nasal disease is often an obstructive disorder, in which the sensory apparatus of the nose remains relatively intact, at least for a certain period of time. People with this condition report that in certain circumstances, either by deliberate manipulation, such as exercise or luck, they can intermittently smell [16]. This may reduce the impact of olfactory loss on the enjoyment of food and hence on any desire to change diet. Second, post-infection smell loss is often associated with olfactory distortion (parosmia; [18]) and hallucinations (phantosmia), and these may be particularly disruptive to food choice [10,21]. Whether this results in eating foods that rely less on olfactory input—such as a Western-style diet—is not known. Head trauma can result in olfactory loss, and in coup contrecoup injury, the olfactory nerve may be severed, and the frontal parts of the brain damaged. Dysexecutive syndrome, with poor impulse control, has previously been linked to weight gain and hyperphagia [22,23]. As poor impulse control is linked to enhanced intake of Western-style foods, traumatic olfactory loss may be linked to greater intake of these foods. A further issue that relates to the cause is the longevity of the impairment. People with congenital olfactory loss may not miss what they never had [8], while rapid onset of olfactory loss (and presentation to clinic) may not allow sufficient time for significant dietary adaptation—although one study found duration of loss not to be linked to food choice [17]. For idiopathic loss, and that from miscellaneous causes, there are, at present, no data.

In the current study we examined a case series of patients with varying diagnoses and degrees of olfactory impairment. Each patient underwent a chemosensory assessment (olfactory, gustatory and retronasal olfaction), in addition to determining the presence of parosmia and phantosmia, and their consumption of a Western-style diet, rich in saturated fat, sugar and salt using a German version [24] of a validated food-frequency measure of these foods [25]. Rather than contrast diet to a healthy control group, we instead examined the association between clinical measures of olfactory performance and the measure of a Western-style dietary pattern. First, we tested for this relationship across the whole sample. Second, we examined this relationship separately for groups of participants who differed in their cause of olfactory impairment.

## 2. Method

### 2.1. Participants

Participants were recruited from successive cases attending the Smell and Taste Outpatients Clinic at the University hospital, Dresden. They were referred to this service if they reported a chemosensory impairment. Patients who had a diagnosed olfactory impairment were eligible to participate and 222 consented to take part. No information about general health was collected, so the sample may contain people with other health conditions.

### 2.2. Procedure

All participants underwent a standard olfactory, gustatory and retronasal olfactory work-up. This involved completing the threshold, discrimination and identification parts of the Sniffin’ Sticks battery [26], the taste sprays test of gustatory function [27] and a test of retronasal olfactory function [28,29]—of which two versions were used (the former out of 3, the latter out of 20), with all scores here presented as percent correct. Duration of the disorder, age and gender, along with presence or absence of parosmia and phantosmia were also obtained. A full diagnosis based on these tests plus an endoscopic examination of the nose and throat and a medical history, were used to establish the aetiology of each person’s olfactory dysfunction. Finally, participants completed the 26 item German version of a scale (called the DFS—dietary fat and sugar scale) to assess intake of discretionary processed foods—a Western-style diet—namely those rich in sugar, salt and saturated fat. Both the German version, and the English version on which it is based, have been validated against more extensive diet diary and food frequency measures of sugar, salt and fat intake [24,25]. The measure has also been shown to negatively correlate with a biomarker of fresh fruit and vegetable intake [30].

### 2.3. Analysis

We undertook a three-step analysis process. First, we examined descriptive statistics, performance against normative data and zero-order correlations for the whole sample, testing for links between a Western-style diet and chemosensory dysfunction. Second, we tested for differences between the aetiology groups using chi-squared and ANOVA across the collected variables (e.g., age, duration, olfactory performance etc.), and then examined relationships between olfactory performance on the Sniffin’ sticks tests and a Western-style diet, using stepwise regression (regression diagnostics indicated no problems with multicollinearity or outliers). This was undertaken in each group where this was possible (i.e., sample size), followed by more specific examinations of each groups’ data. Third, we undertook an exploratory analysis using two of the three subscales of the Western-style diet questionnaire. This questionnaire can produce sweet, fat and sweet-fat subscales, and we focussed on just the single macronutrients sweet and fat (the full scale better measures the combination of sweet and fat) and their relationship to olfactory function in each aetiology group.

## 3. Results

### 3.1. Overall Analyses

Sample descriptive statistics are presented in Table 1. Participants ranged in age from 12 to 85, with approximately half the sample being men. The diagnosed cause of their olfactory loss was classified into six categories, with post-infection and idiopathic aetiologies being the most frequent, and congenital anosmia the least. A small proportion of the sample reported parosmia and/or phantosmia—around 16% and 13%, respectively. Duration of the problem varied between 2 and 204 months, with a duration score not included for the congenitally anosmic participants. As would be expected, olfactory performance on the three components of the Sniffin’ sticks test was abnormal. Using a one-sample *t*-test, with *mu* set conservatively as the normative value for older participants for each test, revealed large impairments on each measure: threshold (normative value = 7.4; *t*(216) = 36.03, *p* < 0.001), discrimination (normative value = 10.6; *t*(214) = 11.10, *p* < 0.001) and identification (normative value = 13.7; *t*(221) = 19.97, *p* < 0.001). For the taste sprays, any score less than 100% (i.e., all 4 correct) is indicative of abnormal taste perception [27]. On this basis 80% of the sample had normal gustation. Of the remaining 20%, 13% identified one taste spray incorrectly, 3% two, 2% three and 1% could not identify any. No normative data were available for the retronasal test (noting it was only completed by half the sample) but we found that it was positively and significantly associated with performance on all three components of the orthonasal Sniffin’ sticks test (*r*’s > 0.35).

We then examined whether there was any significant relationship between the variables in Table 1 and the measure of a Western-style diet. The resultant zero order correlations are presented in Table 2, and there were no significant relationships, except for age, with older participants tending to choose a healthier diet.

### 3.2. Group-Level Analyses

Table 3 presents the same set of demographic variables as Table 1, but now organised by cause of olfactory dysfunction. We started by examining whether there were any differences in these variables between groups. Mean age of the groups significantly varied (*F*(1,216) = 17.55, MSE = 198.8, *p* < 0.001). A Ryan-Einot-Gabriel-Welsh (REGWF) post-hoc range test indicated that congenital anosmics formed a discrete subset, being the youngest, with the remaining groups forming two overlapping subsets, with the idiopathic group being the oldest. There was no significant difference in gender distribution between groups, but it was noted that female participants did tend to be over-represented in the congenital anosmia group. There was a significant group difference in the distribution of parosmia (Chi-squared = 33.50, *p* < 0.0001), with it being most likely in the post-infection group (removing this category eliminated the group difference). There were no group differences in the distribution of phantosmia. Duration also significantly differed (*F*(1,205) = 11.15, MSE = 1581.5, *p* < 0.001)—noting that the congenital group was not included due to the lifelong nature of this condition. An REGWF post-hoc range test indicated that the sino-nasal group formed one homogenous subset, with the longest duration, with the remaining groups forming another. Threshold differed between groups (*F*(5,211) = 4.04, MSE = 3.95, *p* < 0.002), with the REGWF range test indicating three overlapping subsets, with the congenital anosmics poorest and the miscellaneous group the best. Analysis of the discrimination (*F*(5,209) = 3.95, MSE = 9.24, *p* < 0.002) and identification (*F*(1,216) = 6.88, MSE = 14.15, *p* < 0.001) testing, revealed the same group effects as for the threshold data. There were no significant group differences in performance on the retronasal or taste sprays tests or on the Western-style diet score.

We then examined whether relationships between the olfactory performance tests and demographic variables (age, gender and duration of problem) were predictive of a Western-style dietary score using step-wise regression. Regression was not undertaken for the congenital anosmics because of their small sample size, and no significant model emerged for either the post-infection or miscellaneous groups. There were, however, significant models for the idiopathic, sino-nasal disease and trauma groups, which are detailed in Table 4.

**Figure 1:**
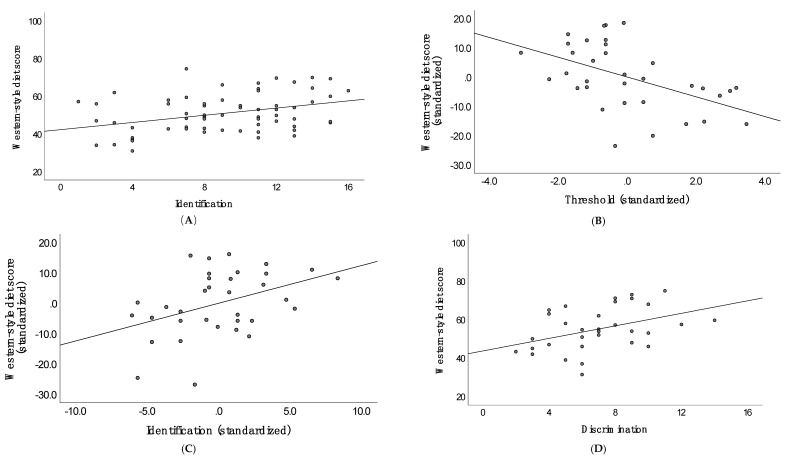
From top left (clockwise)—scatter plot between identification and Western-style diet (WSD) in people with idiopathic olfactory impairment; partial plots of the relationship between threshold, and identification, with WSD in people with sino-nasal disease olfactory impairment; and scatter plot of the relationship between discrimination and WSD in people with olfactory impairment due to trauma (please see Table 4 for associated *r* values). (**A**) Identification, (**B**) Threshold (Standarized), (**C**) Identification (Standarized) (**D**) Discrimination.

For participants with idiopathic olfactory impairment, better performance on the identification test was positively associated with more frequent consumption of a Western-style diet (see Figure 1). For sino-nasal disease, a similar relationship was observed with identification, but for threshold, poorer capacity to detect the test odorant was associated with greater consumption of a Western-style diet (see Figure 1). The zero-order correlations indicated the same pattern of relationships, but partialling out variance attributed to threshold strengthened the positive association with identification (and vice versa). For trauma, better discrimination performance was linked to a greater propensity to consume a Western-style diet.

We then turned our attention to the three groups with no regression output. First, in the post-infection group, they had both one of the shortest duration of symptoms as well as having the highest rate of parosmia. It may be that parosmic participants in this group are more prone to adopt a Western-style diet, but there was no association with the presence or absence of parosmia (*p* = 0.81) in this group.

Second, for the congenital anosmics, there were too few cases to undertake regression, so we just examined zero order correlations between Western-style diet score and the olfactory measures, age and gender. Age (*p* = 0.98) and gender (*p* = 0.64) were not linked to diet, but there was some indication that poorer identification (*r* = −0.56, *p* = 0.096) was associated with a greater propensity for a Western-style diet. There were no associations for discrimination (*p* = 0.43) or threshold (*p* = 0.27) and diet.

Third, for the miscellaneous cause group, there were no associations with any of the other indicators of olfactory abnormality (i.e., parosmia, retronasal performance) and Western-style dietary choices.

### 3.3. Exploratory Analysis of Subcomponents of a Western-Style Diet

Table 5 presents an exploratory analysis of the relationship between two sub-components of a Western-style diet—predominantly sweet but not fatty foods and predominantly fatty but not sweet foods—and olfactory performance, both overall and by group. Yet again, no relationships emerged across the whole sample, but analysis by group revealed a number of putative relationships. In the congenital anosmia group, poorer identification score was linked to higher consumption of savoury fatty foods, but there were no relationships for sugar-rich foods. For the idiopathic group, only sweet foods in general were positively associated with all aspects of olfactory performance (but noting that links to fatty foods had the same sign, although non-significant). There were no significant relationships for the post-infection group. For the sino-nasal disease group, threshold was negatively associated with reported intake of sweet foods—as with fatty foods—but not significantly so. For the trauma group, discrimination was positively associated with sweet food, but no other relationships were significant. In the miscellaneous group, better identification was linked to reduced intake of sweet foods. Finally, only 1/15 of the relationships with fatty foods (by Group) was significant, while 6/15 were for sweet foods—a reliable difference (Fisher exact test, *p* = 0.029).

## 4. Discussion

Given the variability in previous studies examining relationships between olfactory dysfunction and diet, we were not surprised to find that at the aggregate level, choosing to eat more discretionary processed foods, rich in sugar, salt and saturated fat—a Western-style dietary pattern—was not linked to olfactory dysfunction. Examination of these relationships within groups of participants who shared a common aetiology of dysfunction, revealed a different picture, suggesting heterogeneity in this relationship. While we had predicted heterogeneity, its form was not as we had predicted in the Introduction. Patients with idiopathic, trauma and sino-nasal disease-based olfactory dysfunction, all indicated that better olfactory identification or discrimination (and noting that these tests are moderately positively correlated) was linked to greater intakes of a Western-style diet. Sino-nasal disease proved something of an enigma, as here a poorer threshold was linked to greater intake of a Western-style diet, suggesting two different processes operating within this group. For the post-infection group, there was no evidence of any link between olfactory performance and diet. This group—as noted previously [18]—had the highest rate of parosmia, yet this was not linked to a Western-style dietary pattern either. With congenital anosmics, who were by far the youngest group, we found that worse identification performance was associated with greater saturated fat consumption—presumably associated with greater volatile release—and a trend for greater overall consumption of a Western-style diet—but noting the small sample size and that this group should have little if any olfactory capacity (i.e., it could be a chance result). For the miscellaneous group, the same trend was evident as for patients with idiopathic, trauma and sino-nasal disease—better identification here was linked to a lower intake of sweet discretionary foods. Clearly, aetiology has an impact.

Before turning to discuss the possible causes of this heterogeneity in the link between Western-style diet and olfactory dysfunction, it is important to consider whether any other factors may have influenced our findings. A notable departure from previous studies here is using variability within the whole sample, and within each aetiology group, to draw inferences about olfactory dysfunction and diet. With the possible exception of congenital anosmia, which generally involves a complete or near complete loss of olfactory ability, all of the other conditions involved varying degrees of loss, from mild to severe. Essentially, our approach here capitalises on this variability, rather than treating olfactory loss as a unitary phenomenon (i.e., as with group-based comparisons). As we found no mean-based difference in Western-style dietary score between groups, a mean-based contrast with a healthy control group (with little variability in olfactory function) would have been less informative than the correlational approach we adopted. However, it is important to acknowledge that we did not formally contrast the magnitude of the diet-olfaction correlation coefficients between groups. There were two reasons for this. First, it was not immediately obvious which ones should (or could) be compared. Second, and more importantly, as this is the first study to explore these group-based diet-olfaction links, hypothesis generation for future studies was an important goal.

In the idiopathic, sino-nasal disease, trauma and miscellaneous group, there was evidence in each case that better olfactory performance was linked to a poorer diet, namely one adhering more to a Western-style dietary pattern—a pattern that animal data indicate is harmful to brain and physical health by promoting excess food intake [31,32]. There may be several possible explanations for this association. First, Western-style dietary foods are also characterised by their high palatability, making them foods—like chocolate—that are frequently desired [33]. A reduced intake of these foods in people with more severe olfactory impairment could reflect a loss of desire, namely a reduced appetite for such foods because the prospect of eating them is no longer associated with a pleasurable outcome. A second possibility is that it may reflect a conscious decision to switch to a more healthful dietary pattern. The anxiety associated with experiencing a medical condition may provide an incentive to make positive health-related lifestyle changes, including an improved diet [34]. A third possibility is that mild olfactory impairment generates a more potent shift towards a Western-style dietary pattern, than more significant loss. Compensation (i.e., a shift to sweeter, fattier and saltier foods) may have more pay-off for those with a mild impairment than those with a more severe impairment. While these suggestions are all testable, they would presumably apply to anyone with an olfactory loss—but that was not what was observed. In other words, it is necessary to consider other group-based sources of variation that may impinge on these explanations.

Several factors may be important. One is the length of time that the person has experienced olfactory loss for, which might limit the amount of change that could occur if it was short. However, there was no indication that duration had any impact, and a similar conclusion was reached by Postma et al. [17]. A further factor is the presence of parosmia, a feature of the post-infection group. While this was not linked to consumption of a Western-style diet, it may simply be that the presence of parosmia makes eating actively unpleasant, rather than just dull (e.g., [21]). In which case, overall intake reduction may be a more important variable here than diet per se. The enigma of the sino-nasal disease group is also important to consider, as they had two divergent relationships between olfactory function and Western-style diet—pointing to the potential importance of individual olfactory tests. This group may be unique in having some periods of normal olfactory function [16]. It may be that threshold and identification somehow capture who does and does not have the capacity to have periods of normosmia, and this in turn may then affect dietary choices through the types of pathways discussed above. Finally, we noted that where diet was linked to olfactory function, this link (irrespective of sign) was more likely for sweet than for fatty discretionary foods. On the diet questionnaire, many of the purely sweet items are sugar-sweetened beverages, which are an interesting category of nutrient as they are strongly linked to obesity yet are not typically thought of as foods [35]. These may be energy sources that are particularly appealing to people with olfactory loss, something that has not been well explored.

In conclusion, we examined Western-style dietary choices in groups of participants with differing aetiologies of olfactory dysfunction. There was no evidence of a link between a Western-style diet and olfactory dysfunction at the aggregate level, but a group-based approach revealed various patterns, showing both positive and negative associations. We suggest a number of reasons why, in certain cases, greater olfactory dysfunction may be linked to lower intakes of a Western-style diet, and the role that different aetiologies may have in affecting choices for foods that are rich in sugar, salt and fat—characteristics that may appeal following olfactory loss.

## Figures and Tables

**Table 1:** Descriptive statistics of the case series.

Variable	*n*	Mean	Range	SD
Age in years	222	55.6	12–85	16.5
Sex (Male/Female)	222 (95/127)			
Cause of olfactory loss				
Congenital	10			
Idiopathic	66			
Post-infection	63			
Sino-nasal disease	34			
Trauma	33			
Miscellaneous *	17			
Presence of parosmia	35			
Presence of phantosmia	30			
Duration of problem (months)	212	32.9	2–204	43.6
Threshold (Sniffin sticks)	217	2.4	1–8.5	2.1
Discrimination (Sniffin sticks) **	215	8.2	1–15	3.1
Identification (Sniffin sticks) **	222	8.3	0–8	4.0
Retronasal Western test (% correct)	105	67.4	0–100	33.2
Taste sprays (% correct)	220	92.5	0–100	18.0
Western-style diet (DFS *** score)	221	53.2	24–81	10.9

* Iatrogenic, toxin exposure, other diseases. ** Number correct out of 16. *** Dietary fat and sugar scale.

**Table 2:** Zero order correlations for the whole sample between Western-diet score (Dietary fat and sugar scale: DFS) and the other variables.

Variable	DFS Score		
	*n*	*r*	*p*
Age in years	220	−0.19	0.005
Gender	220	0.04	0.51
Presence of parosmia	221	0.09	0.16
Presence of phantosmia	221	−0.05	0.50
Duration of problem	211	−0.07	0.31
Threshold	215	−0.03	0.67
Discrimination	214	0.06	0.40
Identification	220	0.01	0.88
Retronasal test	105	0.06	0.58
Taste sprays	218	0.11	0.12

**Table 3:** Descriptive statistics of the case series by cause of olfactory loss.

Variable	Congenital	Idiopathic	Post-Infection	Sino-Nasal Disease	Trauma	Miscellaneous
Number of cases	10	66	63	34	33	17
Mean age in years (SD)	17.7 (5.6)	61.9 (13.8)	56.2 (15.3)	57.5 (11.4)	53.2 (14.6)	52.6 (17.5)
Percent males	20%	47%	32%	53%	49%	50%
Percent presence of parosmia	0%	8%	37%	0%	18%	6%
Percent presence of phantosmia	0%	18%	11%	9%	21%	6%
Mean duration of problem-months (SD)	-	33.3 (37.7)	19.9 (30.0)	71.9 (65.4)	17.5 (26.9)	27.8 (30.4)
Mean threshold (SD)	1.0 (0.2)	2.7 (2.1)	2.3 (2.0)	2.2 (2.0)	1.6 (1.6)	3.9 (2.7)
Mean discrimination (SD)	5.6 (2.2)	8.6 (3.1)	8.3 (3.1)	8.8 (3.2)	7.0 (2.8)	9.9 (3.2)
Mean identification (SD)	4.8 (1.7)	9.1 (4.0)	8.8 (3.5)	8.1 (4.3)	5.9 (4.0)	11.0 (3.3)
Mean retronasal test (SD)	53.3 (40.2)	68.3 (35.7)	67.6 (34.4)	65.1 (23.7)	65.1 (31.3)	83.3 (35.7)
Mean taste sprays (SD)	92.5 (12.0)	90.0 (22.5)	93.5 (16.3)	93.2 (15.7)	92.3 (18.3)	92.5 (14.7)
Mean Western style diet (SD)	61.1 (6.8)	51.0 (10.3)	52.6 (10.9)	53.5 (11.3)	55.1 (11.0)	54.1 (12.3)

**Table 4:** Stepwise regression analyses, with Western-style diet score as the dependent variable, and age, gender, duration of the problem, identification, discrimination and thresholds scores as predictors.

Variable	ANOVA for Model	*R* ^2^	Predictor Variable/s	*t*(df)	*p*	*r*	Sr	Sr^2^
Idiopathic	*F*(1,60) = 11.77, *p* < 0.001	0.15	Identification	3.43 (60)	0.001	0.41	0.41	0.17
Sino-nasal disease	*F*(2,30) = 5.48, *p* < 0.01	0.22	Threshold	3.25 (30)	0.003	−0.37	−0.51	0.26
			Identification	2.35 (30)	0.026	0.10	0.37	0.14
Trauma	*F*(1,31) = 6.48, *p* < 0.02	0.15	Discrimination	2.55 (31)	0.016	0.42	0.42	0.18

**Table 5:** Exploratory zero-order correlations between two key components of a Western-style diet (Dietary fat and sugar scale: DFS), and measures of olfactory impairment overall, and across the different groups.

Group	Saturated-Fat Rich Foods			Added Sugar Rich Foods		
	Threshold	Identification	Discrimination	Threshold	Identification	Discrimination
Overall	−0.01	−0.02	0.03	0.00	0.01	0.11
Congenital	0.00	−0.77 *	−0.44	−0.08	−0.01	0.07
Idiopathic	0.13	0.19	0.14	0.36 *	0.45 *	0.29 *
Post-infection	0.03	−0.11	−0.08	−0.12	−0.19	0.01
Sino-nasal disease	−0.21	0.19	0.09	−0.34 **	0.06	0.02
Trauma	0.04	−0.11	0.06	−0.01	−0.12	0.37 *
Miscellaneous	−0.01	−0.16	0.45	0.11	−0.47 **	−0.07

* *p* < 0.05; ** *p* < 0.1.
